# The Global Spike: Conserved Dendritic Properties Enable Unique Ca^2+^ Spike Generation in Low-Threshold Spiking Neurons

**DOI:** 10.1523/JNEUROSCI.2740-15.2015

**Published:** 2015-11-25

**Authors:** William M. Connelly, Vincenzo Crunelli, Adam C. Errington

**Affiliations:** ^1^Neuroscience Division, School of Biosciences, and; ^2^Neuroscience and Mental Health Research Institute, School of Medicine, Cardiff University, Cardiff CF24 4HQ, United Kingdom,; ^3^Department of Physiology and Biochemistry, University of Malta, Msida MSD 2080, Malta, and; ^4^Eccles Institute of Neuroscience, The John Curtin School of Medical Research, Australian National University, Canberra City, Australian Capital Territory 2600, Australia

**Keywords:** dendrites, low-threshold spike, T-type Ca^2+^ channel, thalamic reticular nucleus, thalamocortical

## Abstract

Low-threshold Ca^2+^ spikes (LTS) are an indispensible signaling mechanism for neurons in areas including the cortex, cerebellum, basal ganglia, and thalamus. They have critical physiological roles and have been strongly associated with disorders including epilepsy, Parkinson's disease, and schizophrenia. However, although dendritic T-type Ca^2+^ channels have been implicated in LTS generation, because the properties of low-threshold spiking neuron dendrites are unknown, the precise mechanism has remained elusive. Here, combining data from fluorescence-targeted dendritic recordings and Ca^2+^ imaging from low-threshold spiking cells in rat brain slices with computational modeling, the cellular mechanism responsible for LTS generation is established. Our data demonstrate that key somatodendritic electrical conduction properties are highly conserved between glutamatergic thalamocortical neurons and GABAergic thalamic reticular nucleus neurons and that these properties are critical for LTS generation. In particular, the efficiency of soma to dendrite voltage transfer is highly asymmetric in low-threshold spiking cells, and in the somatofugal direction, these neurons are particularly electrotonically compact. Our data demonstrate that LTS have remarkably similar amplitudes and occur synchronously throughout the dendritic tree. In fact, these Ca^2+^ spikes cannot occur locally in any part of the cell, and hence we reveal that LTS are generated by a unique whole-cell mechanism that means they always occur as spatially global spikes. This all-or-none, global electrical and biochemical signaling mechanism clearly distinguishes LTS from other signals, including backpropagating action potentials and dendritic Ca^2+^/NMDA spikes, and has important consequences for dendritic function in low-threshold spiking neurons.

**SIGNIFICANCE STATEMENT** Low-threshold Ca^2+^ spikes (LTS) are critical for important physiological processes, including generation of sleep-related oscillations, and are implicated in disorders including epilepsy, Parkinson's disease, and schizophrenia. However, the mechanism underlying LTS generation in neurons, which is thought to involve dendritic T-type Ca^2+^ channels, has remained elusive due to a lack of knowledge of the dendritic properties of low-threshold spiking cells. Combining dendritic recordings, two-photon Ca^2+^ imaging, and computational modeling, this study reveals that dendritic properties are highly conserved between two prominent low-threshold spiking neurons and that these properties underpin a whole-cell somatodendritic spike generation mechanism that makes the LTS a unique global electrical and biochemical signal in neurons.

## Introduction

Action potential bursts after transient membrane potential hyperpolarization were first described in the thalamus by Andersen and Eccles (1962), a feature they termed “postanodal exhaltation.” Subsequently, after detailed characterization (Deschênes et al., 1984; Jahnsen and Llinás, 1984a,b), the underlying phenomenon has become known as the “low-threshold spike” (LTS) and has been described in cells including subthalamic neurons (Nakanishi et al., 1987), cortical (Goldberg et al., 2004) and striatal (Kawaguchi, 1993) interneurons, serotonergic dorsal raphe neurons (Burlhis and Aghajanian, 1987), neurons of the inferior olivary nucleus (Llinás and Yarom, 1981a), and deep cerebellar nucleus neurons (Llinás and Mühlethaler, 1988).

Nevertheless, LTS are most prominent in glutamatergic thalamocortical (TC) neurons and GABAergic neurons of the thalamic reticular nucleus (TRN) where they are essential for normal thalamic function. LTS play an indispensable role in rhythmic sleep-related activity including delta waves (Steriade et al., 1991), slow (<1 Hz) oscillations (Steriade et al., 1993; Hughes et al., 2002; Blethyn et al., 2006), and 7–14 Hz sleep spindles (Contreras and Steriade, 1996) and also mediate an attention-dependent thalamic “wake-up” signal to the cortex during wakefulness (Swadlow and Gusev, 2001). Moreover, LTS have been implicated in several serious pathological conditions including epilepsy (Pinault et al., 1998), Parkinson's disease (Magnin et al., 2000), and schizophrenia (Ferrarelli et al., 2010).

Recently, Ca^2+^ imaging studies in TC (Errington et al., 2010, 2012; Sieber et al., 2013) and TRN (Crandall et al., 2010; Chausson et al., 2013) neurons demonstrated that LTS are associated with widespread dendritic T-type Ca^2+^ channel-dependent Ca^2+^ influx. Consistent with computational studies (Destexhe et al., 1998; Zomorrodi et al., 2008), these findings imply a central role for dendritic T-type Ca^2+^ channels in LTS generation. However, these data do not explain how the LTS is generated. In fact, three plausible, but distinct, models could be reasoned to explain these results. First, like Na^+^-mediated action potentials (Stuart et al., 1997), LTS might originate in the axo-somatic region and activate dendritic T-type Ca^2+^ channels by active or passive dendritic backpropagation. This mechanism was originally favored for LTS by Llinás and Yarom (1981b) in inferior olivary neurons. Second, like dendritic Ca^2+^ (Schiller et al., 1997) or NMDA (Schiller et al., 2000) spikes, LTS might be locally triggered in dendrites by activation of high-density T-type Ca^2+^ channels before propagating throughout the cell. Third, the electrotonic properties of low-threshold (LT)-spiking neurons mean that, although they are widely distributed in space, T-type Ca^2+^ channels can be simultaneously activated throughout the somatodendritic tree to generate a unique “global spike,” a mechanism suggested for TC neurons in a computational modeling study by Neubig and Sejnowski (2000). Since each has different implications for dendritic signaling in LT-spiking neurons, understanding which mechanism underlies LTS generation is critical. However, to test these hypotheses, knowledge of the dendritic properties of LT-spiking neurons is required, and, unfortunately, this is almost completely absent from our current understanding.

Here, using dual somatodendritic recordings, Ca^2+^ imaging, and computational modeling, we demonstrate that despite differences in morphology and functional roles, TC and TRN neurons have highly conserved dendritic electrical properties. We find that these properties, including highly asymmetric somatodendritic voltage transfer, underpin simultaneous recruitment of spatially distributed T-type Ca^2+^ channels that act in concert to generate a distinctive global Ca^2+^ spike. This unique mechanism, which we predict is shared by all LT-spiking cells, sets the LTS apart from other dendritic electrical and biochemical signals and has important consequences for synaptic signaling, integration and plasticity.

## Materials and Methods

### 

#### 

##### Electrophysiology.

**Figure 1.**
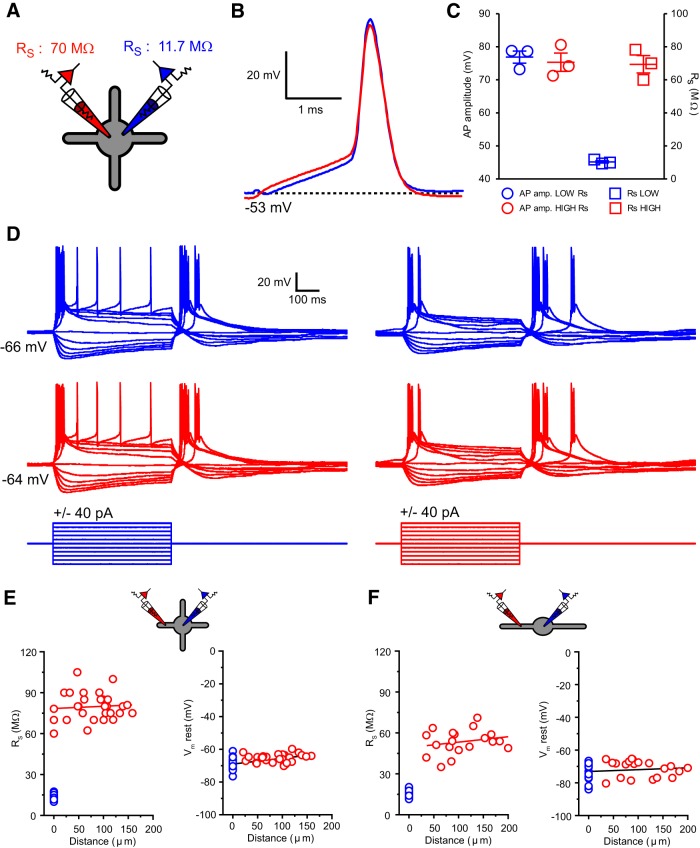
High-resistance recording electrodes accurately record dendritic membrane potential. ***A***, Schematic illustrating the paired somatic recording configuration used to test high-resistance electrode recording fidelity. High-resistance “dendritic” electrodes are shown in red, and low-resistance “somatic” recording pipettes are shown in blue. ***B***, An action potential evoked by current injection through the blue recording electrode is accurately recorded by the red electrode. ***C***, Action potential amplitudes recorded through high-resistance (red) and low-resistance (blue) electrodes are not significantly different. ***D***, Fast and slow voltage changes are recorded faithfully by both high- and low-resistance recording electrodes independently of which electrode injects current (*n* = 3). ***E***, Series resistance (*R*_S_) of dendritic recordings in TC neurons is not significantly correlated with distance from the soma. The dendritic resting membrane potential in TC neurons shows weak distance-dependent depolarization. ***F***, *R*_S_ of dendritic recordings in TRN neurons is not significantly correlated with distance from the soma. The dendritic resting membrane potential in TRN neurons shows weak distance-dependent depolarization.

**Figure 2.**
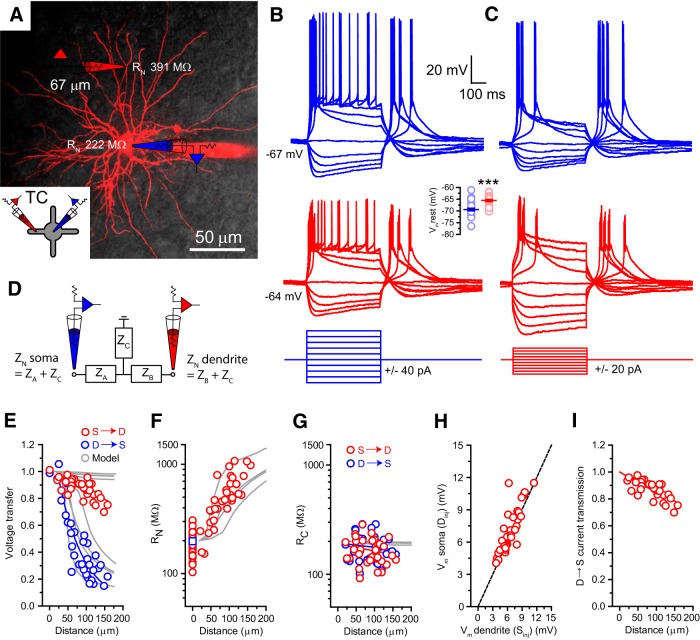
Steady-state electrical properties of TC neuron dendrites. ***A***, Overlay of two-photon fluorescence and infrared scanning gradient contrast images of a dLGN TC neuron showing the location of a somatic (red) and a dendritic (red) recording pipette. Inset, Schematic of the recording configuration illustrating the neuron recorded from (TC) and the placement of recording electrodes. This schematic is used throughout to indicate that the illustrated data are drawn from TC neuron recordings. ***B***, Δ*V*_m_*S*_S_ (blue) and Δ*V*_m_*D*_S_ (red) evoked by 500 ms somatic current injections between −160 and +200 pA. Inset, Mean somatic and dendritic resting membrane potentials. ***C***, Δ*V*_m_*S*_D_ and Δ*V*_m_*D*_D_ evoked by 500 ms dendritic current injections between −100 and +80 pA. ***D***, Schematic illustrating the two-port T-circuit model incorporating the axial impedances Z_A_ and Z_B_ and the common transfer impedance Z_C_ used to describe dendritic electrical conduction. ***E***, Normalized steady-state voltage transfer in the *S*→*D* (red) and *D*→*S* (blue) directions. Solid colored lines, Decaying exponential fits to the experimental data; gray lines, voltage transfer in each direction from four model dendrites. ***F***, Somatodendritic input resistance (*R*_N_) profile. Red circles, Somatic and dendritic *R*_N_ for individual neurons; blue square, mean somatic *R*_N_; gray lines, *R*_N_ profiles of model dendrites. ***G***, Transfer resistance (*R*_C_) versus distance from soma calculated for *S*→*D* (red) and *D*→*S* (blue) directions. Gray lines, *S*→*D R*_C_ for model dendrites. ***H***, Δ*V*_m_*S* and Δ*V*_m_*D* evoked by current injection at the reciprocal recording site. The dashed black line indicates unity. ***I***, Normalized dendrite to soma current transmission versus distance from soma. Solid red line, Decaying exponential fit to the data.

**Figure 3.**
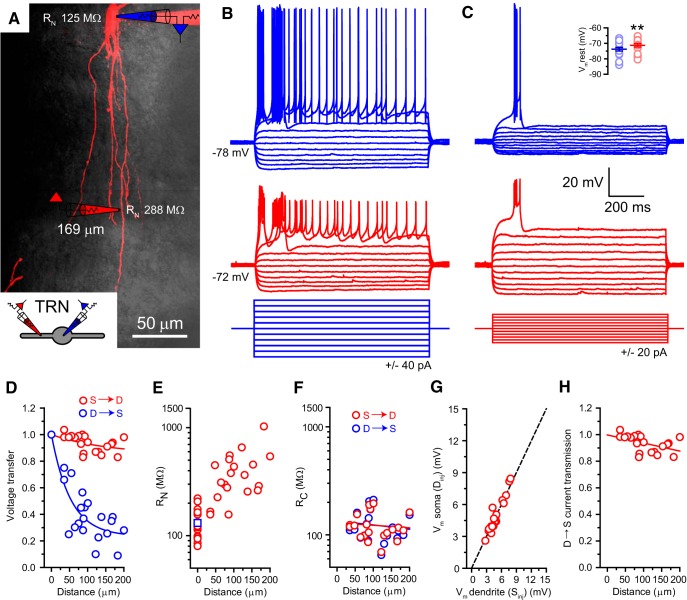
Steady-state electrical properties of TRN neuron dendrites. ***A***, Overlay of two-photon fluorescence and infrared scanning gradient contrast images of a TRN neuron showing the location of somatic (red) and dendritic (red) recording pipettes. Inset, Schematic of the recording configuration illustrating the neuron recorded from (TRN) and the placement of recording electrodes. This schematic is used throughout to indicate that the illustrated data are drawn from TRN neuron recordings. ***B***, Δ*V*_m_*S*_S_ (blue) and Δ*V*_m_*D*_S_ (red) evoked by 500 ms somatic current injections between −200 and +200 pA. ***C***, Δ*V*_m_*S*_D_ and Δ*V*_m_*D*_D_ evoked by 500 ms dendritic current injections between −100 and +100 pA. Inset, Mean somatic and dendritic resting membrane potentials. ***D***, Normalized steady-state voltage transfer in the *S*→*D* (red) and *D*→*S* (blue) directions. Solid colored lines, Decaying exponential fits to the experimental data; gray lines, voltage transfer in each direction from four model dendrites. ***E***, Somatodendritic input resistance (*R*_N_) profile. Red circles, Somatic and dendritic *R*_N_ for individual neurons; blue square, mean somatic *R*_N_; gray lines, *R*_N_ profiles of model dendrites. ***F***, Transfer resistance (*R*_C_) versus distance from soma calculated for *S*→*D* (red) and *D*→*S* (blue) directions. Gray lines, *S*→*D R*_C_ for model dendrites. ***G***, Δ*V*_m_*S* and Δ*V*_m_*D* evoked by current injection at the reciprocal recording site. The dashed black line indicates unity. ***H***, Normalized dendrite to soma current transmission versus distance from soma. Solid red line, Decaying exponential fit to the data.

Coronal slices (250–300 μm) containing the dorsal lateral geniculate nucleus (dLGN) and horizontal slices containing the TRN were prepared from postnatal day 20–25 (P20–P25; dLGN) and P17–P21 (TRN) Wistar rats of either sex, which were deeply anesthetized using isoflurane, as described by Errington et al. (2010) and with approval of the Cardiff University Research Ethics Committee and in accordance with the Home Office Animals (Scientific Procedures) Act 1986, United Kingdom. For recording, slices were transferred to a submersion chamber continuously perfused with warmed (33–4°C) aCSF [in mm: 125 NaCl, 2.5 KCl, 2 CaCl_2_, 1 MgCl_2_, 1.25 NaH_2_PO_4_, 25 NaHCO_3_, and 25 d-glucose (305 mOsm)] at a flow rate of 2.5–3 ml/min. Somatic whole-cell patch-clamp recordings were made from TC and TRN neurons (visually identified by infrared gradient contrast video microscopy) using a Multiclamp 700B amplifier (Molecular Devices) and pipettes with resistances of 4–6 MΩ when filled with an internal solution containing (in mm) 130 K-gluconate, 20 KCl, 10 HEPES, 0.16 EGTA, 2 Mg-ATP, 2 Na_2_-ATP, and 0.3 Na_2_-GTP, pH 7.3 (295 mOsm), and supplemented with 50 μm Alexa 594 (Invitrogen). Recording solutions did not routinely include any synaptic blocking drugs or other blocking toxins unless specifically indicated. Electrophysiological data were sampled at 20–50 kHz and filtered at 6 kHz. Somatic series resistance at the start of experiments was between 9 and 15 MΩ and varied ≤20% during recordings. Two-photon fluorescence microscopy, using a Prairie Ultima (Prairie Technologies) microscope and titanium/sapphire pulsed laser (Chameleon Ultra II; Coherent) tuned to λ = 810 nm, was combined with infrared scanning gradient contrast to make targeted dendritic patch-clamp recordings from thin (∼0.7–2 μm) dendrites of TC (see Fig. 2*A*) and TRN (see Fig. 3*A*) neurons. Dendritic recording pipettes were made from borosilicate glass capillaries (BF200-100-10; Sutter Instruments) and had resistances of 25–40 MΩ when filled with the internal recording solution described above. This resulted in recordings that had high series resistance values (TC: 60–105 MΩ, 79.6 ± 1.8 MΩ, *n* = 28; TRN: 35–71 MΩ, 53.5 ± 2.0 MΩ, *n* = 20). However, series resistance of dendritic recordings was not correlated with distance of the recording site from the soma in either TC (*n* = 28, *r* = 0.07, *p* = 0.70, Pearson's *r* test; Fig. 1*E*) or TRN (*n* = 20, *r* = 0.22, *p* = 0.36, Pearson's *r* test; Fig. 1*F*) neurons. Nonetheless, since the recording pipette behaves as a low-pass filter with a cutoff frequency inversely to proportional to its resistance (*R*_p_) and capacitance (*C*_p_), it is critical that careful compensation is performed so that high-frequency dendritic voltages are minimally distorted. Thus, dendritic and somatic bridge balance and pipette capacitance neutralization were carefully adjusted and monitored throughout experiments by application of low-frequency (50 Hz), low-amplitude current steps (10–40 pA). Importantly, in our recordings, although the low capacitance and high input resistance of the dendrites resulted in large and rapid dendritic voltage responses, these were considerably slower than the near instantaneous voltage drop observed with an “unbalanced bridge.” Nonetheless, absolutely “perfect” bridge balance may not have been achieved in each recording. However, potential small errors in bridge balance settings cannot account for the dendritic voltage responses and input resistances we measure (see Results). We tested the ability of high-resistance electrodes to record membrane potential transients by performing dual somatic recordings from TC neurons with high-resistance (*R*_S_: 69.4 ± 5.3 MΩ, *n* = 3) and low-resistance (*R*_S_: 10.3 ± 0.7 MΩ, *n* = 3; Fig. 1*A–C*) electrodes. We found no significant (*p* > 0.49, paired *t* test) difference in the amplitude of action potentials recorded by high-resistance electrodes versus low-resistance electrodes (Fig. 1*B*,*C*), consistent with previous findings (Nevian et al., 2007; Bathellier et al., 2009; Larkum et al., 2009; Krueppel et al., 2011). Thus, we conclude that compensation can be performed accurately even for high-resistance electrodes and that the dendritic voltages we report are accurate.

##### Measurement of steady-state electrotonic parameters.

To measure the steady-state voltage and current transfer properties of dendrites, long current injection steps were used (500–1000 ms). Current was injected at either the somatic or dendritic recording electrode resulting in four possible voltage measurements, namely the voltage at the soma with somatic injection (VS_S_), voltage at the dendrite with somatic injection (VD_S_), voltage at the soma with dendritic injection (VS_D_), and voltage at the dendrite with dendritic injection (VD_D_). Local somatic and dendritic input resistance (*R*_N_) was measured by dividing the average (over 50 ms) local voltage response at the end of the step by the injected current (*R*_N_ soma = VS_S_/*I*_S_ and *R*_N_ dendrite = VD_D_/*I*_D_). The transfer resistance (*R*_C_) was measured by dividing the voltage response at the noncurrent injecting electrode by the current injected at the reciprocal electrode (*R*_C_ = VS_D_/*I*_D_ or VD_S_/*I*_S_). Steady-state voltage transfer between soma and dendrites (VS_S_→VD_S_) was defined by VD_S_/VS_S_ and between dendrites and soma (VD_D_→VS_D_) by VS_D_/VD_D_. Current transmission was calculated as the ratio of current arriving at the soma to the current injected into the dendrite. According to Ohm's law, the current arriving at the soma is equal to the somatic voltage response to current injected in the dendrite divided by the somatic *R*_N_. Therefore, the current transmitted to the soma is *I*_S_ = VS_D_/*R*_N_ = VS_D_/VS_S_. To quantify the relationship between measured values (e.g., steady-state voltage attenuation) and the distance between the dendritic and somatic recording electrodes, data were fit with a mono-exponential function, *f*(*x*) = exp(−*x*/λ_eff_), to yield the “effective space constant” λ_eff_. Approximations of the electrotonic lengths (*L*) of TC and TRN neuron dendrites were calculated based on the space constant (λ_eff_) derived from decaying exponential fits to our dendritic recording data (*L* = physical dendritic length/λ_eff_). Estimates of the mean physical lengths of TC (160 μm) and TRN (400 μm) neuron dendrites were based on previous detailed anatomical measurements made by Ohara and Havton (1994; TC), Spreafico et al. (1991; TRN), and Lübke (1993; TRN).

Low-threshold spikes were evoked by injection of depolarizing (40–120 pA) and/or hyperpolarizing (−80 to −200 pA) current steps (1 s duration) into the somatic and dendritic recording electrodes in TC and TRN neurons held at their resting membrane potential. In TC neurons, rebound bursts were evoked by hyperpolarizing the soma from the resting membrane potential to −82.5 ± 1.5 mV (*n* = 28). In TRN, cells were held at −55 mV and hyperpolarized to −70.3 ± 1.4 mV (*n* = 20). For TC neurons, the peak amplitude was measured as the difference between the peak of the Ca^2+^ spike component of the LTS and the resting membrane potential immediately before LTS were evoked (see Fig. 6*A*). In TRN neurons, all quantitative data are derived from measuring the peak of the Ca^2+-^spike component of the LTS in TTX (0.5 μm) and the resting membrane potential. Although we evoked LTS using both hyperpolarizing (from rest in TC neurons and from −55 mV in TRN neurons) and depolarizing current steps, quantitative data are derived only from depolarizing currents. LTS threshold was determined as the point at which the first derivate (δ*V*/δ*t*) of the membrane potential resulting from the minimum depolarizing current step required to generate an LTS exceeded 0.5 mV · ms^−1^.

##### Frequency-dependent impedance.

Impedance (Z) was obtained by injecting a sinusoidal frequency-modulated current (*I*_CHIRP_) of linearly increasing frequency (0.1–20 Hz) and 10 s duration into either the somatic or dendritic recording pipette. *I*_CHIRP_ was calculated by the following:

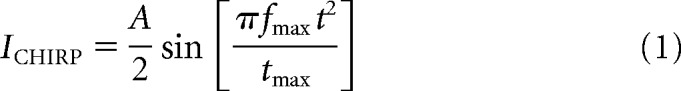
 where *A* is the peak-to-peak amplitude, *f*_max_ is the peak frequency reached at time *t*_max_, and *t* is time (starting at zero). Fast Fourier transforms (FFTs) of the time domain current and voltage traces produced complex numbers comprising real, Re(*f*), and imaginary, Im(*f*), parts that represent the signal in the frequency domain. Subsequently, the impedance magnitude was calculated by the following:

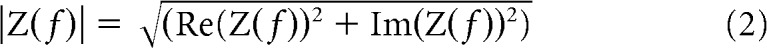
 and the impedance phase (in radians) was calculated by the following:

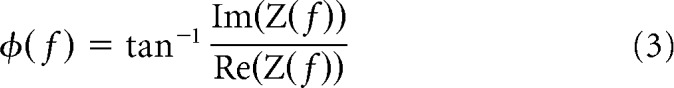
 Somatic and/or dendritic input impedance amplitude profiles (ZAP_N_) were calculated by dividing the magnitude of the local voltage response [Z(*f*)_local voltage_] by the magnitude of the local current injection [ZAP_N_ = Z(*f*)_local voltage_/Z(*f*)_local current_]. The amplitude of *I*_CHIRP_ (±20 pA) was adjusted to keep voltage responses below the threshold for LTS initiation, and the lowest frequency analyzed was 0.5 Hz to avoid boundary artifacts of the FFT (spectral resolution, 0.15 Hz). Transfer impedance amplitude profiles (ZAP_C_) were calculated by injecting *I*_CHIRP_ into the somatic or dendritic recording electrode and recording the voltage response at the reciprocal electrode (*V*_reciprocal_). Thus, ZAP_C_ = Z(*f*)_reciprocal_ _voltage_/Z(*f*)_local current_. Input impedance phase profiles (ZPP_N_) were calculated as the algebraic difference between the phase of the local voltage response [Φ(*f*)_local voltage_] and the local current input [Φ(*f*)_local current_]. Transfer impedance phase profiles (ZPP_C_) were calculated as the algebraic difference between phase of the local injected current [Φ(*f*)_local current_] and the phase of the voltage at the reciprocal electrode [Φ(*f*)_reciprocal voltage_]. All voltages represent averages of five trials.

Somatic ZAP_N_ were fit with a single-pole resistance and capacitance transfer function of the following form:

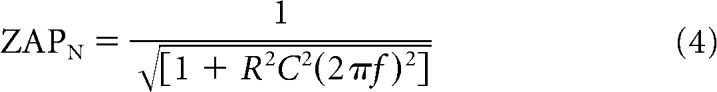
 where *R* is resistance (constrained to fit the mean somatic steady-state *R*_N_), *C* is capacitance, and *f* is frequency. The cutoff frequency (*f*_c_) is calculated as follows:

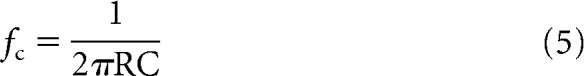


##### Two-photon *Ca*^2+^ imaging.

Two-photon Ca^2+^ imaging and focal TTA-P2 application were performed using a Prairie Ultima microscope (Prairie Technologies) and titanium/sapphire pulsed laser (Chameleon Ultra II; Coherent) tuned to λ = 810 nm as previously described in detail by Errington et al. (2010). Briefly, the internal patch pipette solution, described above, was supplemented with the red Ca^2+^-insensitive dye Alexa Fluor 594 (25 μm) and the green Ca^2+^ indicator Fluo 5F (300 μm), and cells were allowed to fill for 20 min before commencing experiments. Line scans were performed at 500 Hz (2 ms per line) at selected dendritic regions of interest, and the ratio of green over red fluorescence (Δ*G*/*R*) was measured as the stimulus-evoked change in intracellular Ca^2+^.

##### Computational modeling.

Simulations were performed using NEURON 7.2 (Carnevale and Hines, 2006). A morphologically accurate model cell was taken from the study by Briska et al. (2003). Briefly, they prepared brain slices (500 μm) from adult Sprague Dawley rats (100–300 g), and a biocytin-filled TC neuron from the dLGN was reconstructed using the Neurolucida system (see Fig. 9*A*). This was subsequently used to produce a multicompartmental NEURON model comprising 196 sections with a total of 684 segments. We obtained passive biophysical properties by fitting the model cell to the parameters that gave the best fit to our somatic and dendritic recordings using the principal axis method (PRAXIS). Initially, axial resistance (*R*_i_) and specific membrane capacitance (*C*_m_), along with a rough approximation of membrane conductance, were estimated using purely passive conditions. Fits were made to somatic and dendritic voltage transients obtained during dual physiological recordings in response to 3 ms, −100 pA current injections steps. This approach yielded best-fit estimates for *R*_i_ of 170 Ω · cm and for *C*_m_ of 0.7 μF/cm^2^. Leak conductance (*g*_LEAK_) and hyperpolarization-activated cyclic nucleotide-gated conductance (H-current, *g*_H_) were then estimated by fitting the model to voltages evoked by longer 500 ms current steps of −40 pA. Several different models of *g*_H_ were tested, but our recordings were best described by the rate of *g*_H_ activation in the model proposed by McCormick and Pape (1990). Fitting was attempted with linear variation of the density of these conductances along the somatodendritic axis. The data were, however, found to be best fit with an invariant channel density, a finding that is consistent with previous *in vitro* findings (Williams and Stuart, 2000). Several models of T-type Ca^2+^ channels were investigated, and the best fit to our data was obtained using the model proposed by Destexhe et al. (1998). The gating of the T-type Ca^2+^ channel was shifted by 12 mV in the hyperpolarizing direction. This was necessary to account for differences in recording conditions between our study, where a liquid junction potential of 12 mV was not corrected for, and the study by Huguenard and Prince (1992), which obtained the experimental data used by the model of Destexhe et al. (1998). Variations in somatodendritic T-type conductance distribution were tested. When channels were restricted to the soma and/or proximal dendrites, the intracellular dendritic calcium transients (ΔCa^2+^) previously reported (Crandall et al., 2010; Errington et al., 2010, 2012; Sieber et al., 2013) could not be reproduced. However, using a uniform somatodendritic channel density distribution produced the best fit to electrophysiological recordings as well as producing the roughly uniform ΔCa^2+^ described in our previous study. To describe intracellular Ca^2+^ accumulation and removal, a simple Ca^2+^ extrusion mechanism was inserted. This model was described by McCormick and Huguenard (1992) and had first-order kinetics leading to extrusion of Ca^2+^ to a final resting concentration of 200 nm, with a time constant of 5 ms. A Ca^2+^-activated cation channel (*g*_CAN_) was included in our model using values calculated by Destexhe et al. (1994). Fast voltage-gated sodium channels (*g*_Na_) were included from the study by Destexhe et al. (1998). Finally, an inwardly rectifying potassium channel (*g*_KIR_) was required to accurately reproduce the steady-state *I–V* behavior observed during dual recordings. The model used is described by Williams et al. (1997), although we found that a shift of 15 mV in the depolarizing direction was required to allow the model to match our electrophysiological data. Once the necessary channels were inserted into the model, we recalculated *g*_LEAK_, by fitting to electrophysiological data, to account for their conductance at rest.

Thus, our final model comprised the following: *g*_LEAK_ was modeled with a reversal potential of −79 mV and conductance of 150 μs/cm^2^, and *g*_H_ had a reversal potential of −45 mV and peak conductance of 150 μs/cm^2^. T-type Ca^2+^ channels (*g*_T_) were modeled with a reversal potential of 120 mV and a permeability of 0.7 μm/s. The *g*_CAN_ had a conductance of 250 μS/cm^2^ and reversal potential of −20 mV. The *g*_KIR_ and the fast voltage-gated potassium channel (*g*_Kf_) had conductances of 20 μs/cm^2^ and 50 mS/cm^2^, respectively. Both potassium channels had reversal potentials of −100 mV. The *g*Na had conductance of 50 mS/cm^2^ and reversal potential of 50 mV. Throughout this manuscript, when referring to the ability of *g*_T_ to provide current, we use the term “conductance” rather than “permeability.” Whereas the model is in actuality based on permeability rather than conductance, we use this naming convention to simplify the text.

Simulations were solved with a fixed time step of 50 μs or using the implicit variable time-step solver CVODE, depending on the nature of the simulation (Cohen and Hindmarsh, 1996). Dendrites used for simulations were selected based on their morphology. In most model dendrites, overall dendritic lengths were very similar. In comparison, the length of the primary dendrites varied, and thus the higher-order thin dendrites also had different dendritic path lengths. Primary dendrites, because of their relatively large diameter, added little axial resistance, and thus distal dendrites arising from longer first-order dendrites had lower input resistance than those arising from shorter primary dendrites. Thus, we selected four dendrites with input resistance profiles that encompassed the range we found in our experiments (see Fig. 9*A*).

##### Data analysis and statistics.

Data analysis was performed using pClamp 10 (Molecular Devices), Matlab (Mathworks), ImageJ (NIH), and Prism (GraphPad Software) software. Distance measurements were performed on image stacks collected at the end of recordings using MetaMorph (Molecular Devices) as described by Errington et al. (2010). Statistical testing was by paired/unpaired *t* test, repeated-measures ANOVA, or Pearson's *r* test where appropriate. Space constants were derived from exponential fits to the experimental data. All values are given as mean ± SEM.

## Results

### Conserved steady-state electrical properties between TC and TRN neuron dendrites

Previous studies indicate that the majority of T-type Ca^2+^ channels responsible for generating LTS in LT-spiking neurons are found in their dendrites (Destexhe et al., 1998; Williams and Stuart, 2000; Crandall et al., 2010; Errington et al., 2010). Therefore, to investigate the mechanism underlying LTS generation, we first characterized the basic electrotonic properties of LT-spiking cells that, unlike those of other cell types, remain almost wholly unknown. We combined two-photon fluorescence with infrared scanning gradient contrast (see Materials and Methods) to obtain, for the first time, recordings along the entire somatodendritic axis of TC neurons (range, 20–158 μm; mean distance, 83.2 ± 6.7 μm; *n* = 35; Fig. 2*A*) and along almost the entire length of TRN neuron dendrites (range, 35–200 μm; mean distance, 105.4 ± 11.12 μm; *n* = 20; Fig. 3*A*). In our recordings, we observed a small, distance-dependent dendritic resting membrane potential depolarization relative to the soma in both TC (slope, 0.035 mV · μm^−1^; *n* = 35; *r* = 0.53; *p* < 0001, Pearson's *r* test; Fig. 1*E*) and TRN (slope, 0.011 mV · μm^−1^; *n* = 20; *r* = 0.14; *p* = 0.039, Pearson's *r* test; Fig. 1*F*) neurons. In both cell types, the mean dendritic resting membrane potential was significantly more depolarized than at the soma (TC: S, −73.8 ± 1.2 mV; D, −71.3 ± 1.1 mV; *n* = 20; *p* = 0.0019, paired *t* test; Fig. 3*C*; TRN: S, −69.4 ± 0.6 mV; D, −65.5 ± 0.5 mV; *n* = 35; *p* < 0.0001, paired *t* test; Fig. 2*B*).

We tested the steady-state (DC) electrical conduction properties of these neurons using long, stepwise current injections. At rest, somatic current injection produced somatic voltage responses (VS_S_) typical of TC (Fig. 2*B*) and TRN (Fig. 3*B*) neurons, having prominent LTS. In both neurons, the concurrent dendritic voltage responses (VD_S_) were strikingly similar to VS_S_ [TC (Fig. 2*B*), TRN (Fig. 3*B*)] regardless of the dendritic recording location. In contrast, dendritic current injection produced VD_D_ that were increasingly larger than VS_D_ as the dendritic recording site became more distal [TC (Fig. 2*C*), TRN (Fig. 3*C*)]. Steady-state voltage transfer was evaluated by comparing somatic and dendritic voltage responses produced by −40 pA hyperpolarizing current steps. Somatic current injections revealed highly efficient steady-state voltage transfer between soma and dendrites (VS_S_→VD_S_) in both TC (range, 0.69–1.01; 0.88 ± 0.01; *n* = 35; Fig. 2*E*) and TRN (range, 0.83–1.04; 0.93 ± 0.01; *n* = 20; Fig. 3*D*) neurons. Fitting decaying exponential functions to the data yielded effective space constants of λ_eff_ = 654 μm (TC; Fig. 2E) and λ_eff_ = 1521 μm (TRN; Fig. 3*D*). Using previous measurements of dendritic length (Spreafico et al., 1991; Ohara and Havton, 1994; Lübke, 1993), we estimated the electrotonic length of these dendrites. In the somatofugal direction, both TC (*L* ≈ 0.24) and TRN (*L* ≈ 0.26) neurons have similar short electrotonic lengths and are, therefore, very electrotonically compact. In clear comparison, steady-state voltage transfer between dendrites and soma (VD_D_→VS_D_) was much less efficient in TC (0.15–1.05, 0.45 ± 0.05, λ_eff_ = 69 μm, L ≈ 2.3, *n* = 35; Fig. 2*E*) and TRN (0.09–0.75, 0.38 ± 0.04, λ_eff_ = 98 μm, L ≈ 4.1, *n* = 20; Fig. 3*D*) neurons. Interestingly, asymmetry between (VS_S_→VD_S_) and VD_D_→VS_D_ was remarkably similar in both neurons despite their differing dendritic morphology. Such asymmetry is consistent with cable theory of branching neurons and is attributable to imbalances in impedance load encountered by current flowing in opposing directions along the dendritic cable as a result of the significantly different boundary conditions imposed by the soma/proximal dendrites and sealed terminal ends of the dendrite (Rall and Rinzel, 1973; Carnevale et al., 1997; Jaffe and Carnevale, 1999). Consistent with this concept, dendritic input resistance (*R*_N_) increased markedly in both TC (156.6 MΩ at 25 μm to 1.1 GΩ at 114 μm; 537.5 ± 41.0 MΩ, *n* = 35; Fig. 2*F*) and TRN (157 MΩ at 35 μm to 1.03 GΩ at 183 μm; 388.8 ± 46.2 MΩ, *n* = 20; Fig. 3*E*) neurons as a function of distance from the soma (TC somatic *R*_N_: 102.9–450.3 MΩ, 197.6 ± 11.4 MΩ, *n* = 35; Fig. 2*F*; TRN somatic *R*_N_: 80.0 to 221.3 MΩ, 131.0 ± 8.4 MΩ, *n* = 20; Fig. 3*E*). In TC neurons, these experimentally measured *R*_N_ values are consistent with those previously predicted by computational modeling studies (Lajeunesse et al., 2013). Significantly, however, in both types of LT-spiking neurons, unlike steady-state voltage transfer between dendrite and soma, current transmission, calculated as the ratio of current arriving at the soma to that injected into the dendrite, was highly efficient (TC: λ_eff_ = 614 μm, *n* = 35; Fig. 2*I*; TRN: λ_eff_ = 1519 μm, *n* = 20; Fig. 3*H*; Carnevale and Johnston, 1982). Consequently, the majority of current injected into the dendrite flows along the axial path toward the soma, meaning the observed dendrite to soma voltage attenuation is caused by large differences in local dendritic and somatic *R*_N_ rather than substantial current loss through a “leaky” dendritic tree.

In dendrites of both LT-spiking neurons, VS_D_ and VD_S_ were nearly identical (“reciprocity”), indicating linear behavior at subthreshold voltages (Koch, 1984). Linear fits of VS_D_ versus VD_S_ revealed slopes of 1.06 ± 0.03 in TC (*n* = 35; Fig. 2*H*) and 1.01 ± 0.02 in TRN (*n* = 20; Fig. 3*G*) neurons. Consequently, electrical conduction between soma and dendrites is well described by a linear two-port circuit model (Carnevale and Johnston, 1982; Jaffe and Carnevale, 1999). Briefly, two-port electrotonic theory proposes that electrical coupling between any two points in a linear system, i.e., the dendrite and soma, can be described by an equivalent circuit consisting of three impedances (or resistances in the DC case), two axial impedances, and a common transfer impedance (Z_C_; Fig. 2*D*; Jaffe and Carnevale, 1999). This model predicts that in neurons displaying steep somatodendritic gradients of *R*_N_, as described above for TC (Fig. 2*F*) and TRN (Fig. 3*E*) neurons, to account for the observed VD_D_→VS_D_ voltage attenuation, transfer resistance (*R*_C_) will vary little with distance from the soma (Jaffe and Carnevale, 1999). This is precisely what we observed in our somatodendritic recordings [TC (Fig. 2*G*), TRN (Fig. 3*F*)]. Moreover, as predicted for a linear system, measured *R*_C_ values were equal whether calculated from somatic (VD_S_) or dendritic (VS_D_) current injections in both TC (VD_S_ *R*_C_, 165.2 ± 7.8 MΩ; VS_D_ R_C_, 177.3 ± 8.9 MΩ; *n* = 35, *p* = 0.07, paired *t* test; Fig. 2*G*) and TRN (VD_S_ R_C_, 122.2 ± 7.8 MΩ; VS_D_ R_C_, 122.7 ± 8.9 MΩ; *n* = 20, *p* = 0.85, paired *t* test; Fig. 3*F*) neurons. This distance-dependent profile of *R*_c_ in LT-spiking neurons demonstrates that currents of equal size entering any location within the dendritic tree will be almost equally efficient in depolarizing the soma and produce similar changes in somatic voltage. The steady-state electrical conduction properties of both TC and TRN dendrites were similar when the neurons were depolarized (−55 mV) as they were at rest (Table 1). Thus, despite having very different dendritic morphologies, the steady-state electrotonic properties of TC and TRN neurons are remarkably similar.

**Table 1.** Somatodendritic electrical properties of TC and TRN neurons at −70 and −55 mV

	TC		TRN	
	−70 mV	−55 mV	−70 mV	−55 mV
Input resistance (*R*_N_) soma (MΩ)	197.6 ± 11.4	305.1 ± 15.8	131.0 ± 8.4	179.2 ± 24.3
Input resistance (*R*_N_) dendrite (MΩ)	537.5 ± 41.0	679.9 ± 93.5	388.8 ± 46.2	441.1 ± 53.9
Transfer resistance (*R*_C_) *S*→*D* (MΩ)	165.2 ± 7.8	272.0 ± 23.0	122.2 ± 7.8	169.5 ± 22.6
Transfer resistance (*R*_C_) *D*→*S* (MΩ)	177.3 ± 8.9	286.5 ± 13.4	122.7 ± 8.9	168.6 ± 21.4
Reciprocity slope	1.06 ± 0.03	1.04 ± 0.05	1.01 ± 0.02	0.98 ± 0.02
τ_eff_ *S*→*D* (μm)	654	816.8	1521	2051
τ_eff_ *D*→*S* (μm)	69	116.2	98	105.1
*n*	35	5	20	13

Data are presented as mean ± SEM. The numbers of recorded dendrites (*n*) are as indicated.

### Frequency-dependent impedance properties of LT-spiking neurons

**Figure 4.**
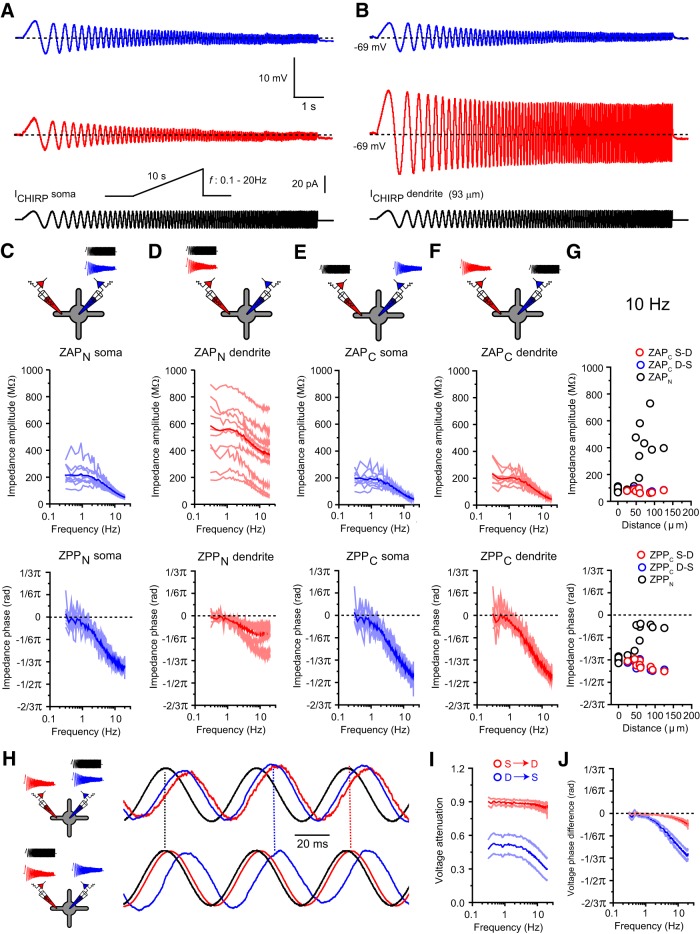
Frequency-dependent properties of TC neuron dendrites. ***A***, Example traces of somatic (*V*_CHIRP_*S*, blue) and dendritic (*V*_CHIRP_*D*, red) voltage responses to somatic frequency-modulated current injections (*I*_CHIRP_*S*, black). ***B***, As in ***A*** for dendritic current injections (*I*_CHIRP_*D*). ***C***, Plots of somatic ZAP_N_ and ZPP_N_. Light blue lines, Individual neurons; dark blue line, mean somatic ZAP_N_/ZPP_N_. ***D***, Plots of dendritic ZAP_N_ and ZPP_N_. Light red lines, Individual neurons; dark red line, mean dendritic ZAP_N_/ZPP_N_. ***E***, Plots of somatic ZAP_C_ and ZPP_C_. Light blue lines, Individual neurons; dark blue line, mean somatic ZAP_C_/ZPP_C_. ***F***, Plots of dendritic ZAP_C_ and ZPP_C_. Light red lines, Individual neurons; dark red line, mean somatic ZAP_C_/ZPP_C_. ***G***, Input impedance amplitude (top) and phase (bottom; black circles, ZAP_N_/ZPP_N_) and *S*→*D* (red circles) and *D*→*S* (blue circles) transfer impedance amplitude and phase (ZAP_C_/ZPP_C_) versus distance from soma for 10 Hz current injections. ***H***, Example traces depicting the phase relationship between voltage and injected current (black) at 20 Hz for somatic (blue) and dendritic (125 μm, red) recordings. ***I***, Plot of frequency-dependent voltage attenuation between *S*→*D* (red) and *D*→*S* (blue) versus distance from soma. ***J***, Plot of frequency-dependent voltage phase difference between *S*→*D* (red) and *D*→*S* (blue) versus distance from soma.

**Figure 5.**
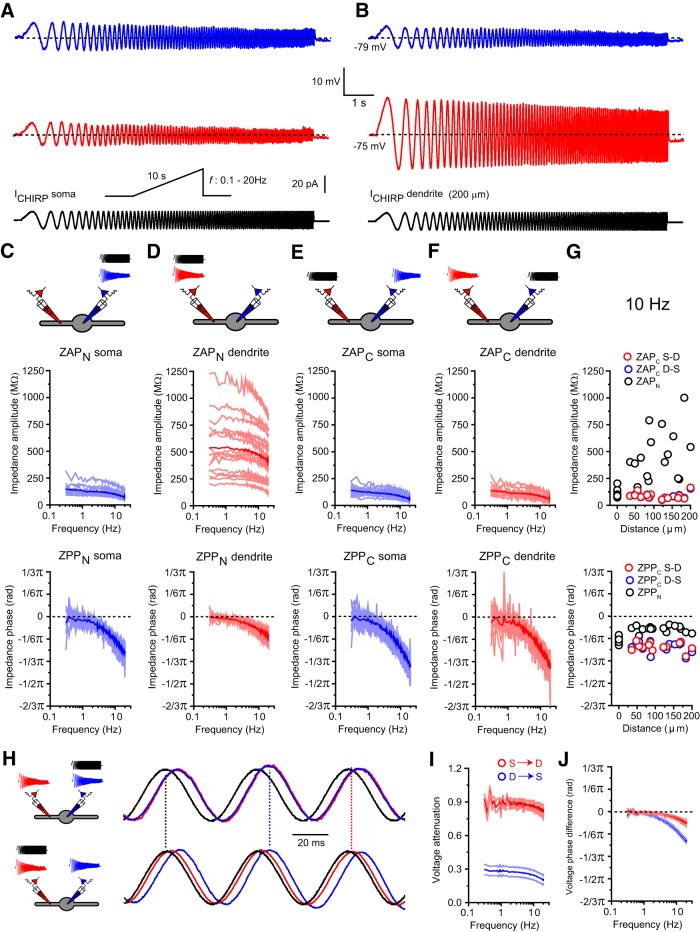
Frequency-dependent properties of TRN neuron dendrites. ***A***, Example traces of somatic (*V*_CHIRP_*S*, blue) and dendritic (*V*_CHIRP_*D*, red) voltage responses to somatic frequency-modulated current injections (*I*_CHIRP_*S*, black). ***B***, As in ***A*** for dendritic current injections (*I*_CHIRP_*D*). ***C***, Plots of somatic ZAP_N_ and ZPP_N_. Light blue lines, Individual neurons; dark blue line, mean somatic ZAP_N_/ZPP_N_. ***D***, Plots of dendritic ZAP_N_ and ZPP_N_. Light red lines, Individual neurons; red line, mean dendritic ZAP_N_/ZPP_N_. ***E***, Plots of somatic ZAP_C_ and ZPP_C_. Light blue lines, Individual neurons; blue line, mean somatic ZAP_C_/ZPP_C_. ***F***, Plots of dendritic ZAP_C_ and ZPP_C_. Light red lines, Individual neurons; red line, mean somatic ZAP_C_/ZPP_C_. ***G***, Input impedance amplitude (top) and phase (bottom; black circles, ZAP_N_/ZPP_N_) and *S*→*D* (red circles) and *D*→*S* (blue circles) transfer impedance amplitude and phase (ZAP_C_/ZPP_C_) versus distance from soma for 10 Hz current injections. ***H***, Example traces depicting the phase relationship between voltage and injected current (black) at 20 Hz for somatic (blue) and dendritic (125 μm, red) recordings. ***I***, Plot of frequency-dependent voltage attenuation between *S*→*D* (red) and *D*→*S* (blue) versus distance from soma. ***J***, Plot of frequency-dependent voltage phase difference between *S*→*D* (red) and *D*→*S* (blue) versus distance from soma.

So far, we have examined the electrical properties of TC and TRN neuron dendrites under steady-state (DC) conditions. Of course, neuronal signals including LTS are dynamic and time-varying signals, and TC and TRN neurons are known to reside in active oscillating circuits. Electrical impedance is the ratio of the recorded voltage response to an injected current and is a standard measurement for characterizing frequency-dependent neuronal membrane properties. Impedance is a complex quantity having a real part, the resistance, and an imaginary part, the reactance. Whereas resistance (*R*) is always a positive value, reactance can be either a positive or negative value depending on the presence of capacitive (*C*) or inductive (*L*) elements within the circuit (Narayanan and Johnston, 2008). The phase relationship between injected current and recorded voltage is a measure of reactance and can be used to determine the presence and function of inductive elements within neurons. Therefore, we tested frequency-dependent properties by injecting sinusoidal frequency-modulated currents (*I*_CHIRP_, 0.1–20 Hz, ±20 pA, 10 s) into the soma (*I*_CHIRP_*S*) and dendrites (*I*_CHIRP_*D*) of these two types of LT-spiking neurons [TC (Fig. 4*A*,*B*), TRN (Fig. 5*A*,*B*)].

First, using the discrete Fourier transforms of the somatic (*V*_CHIRP_*S*) and dendritic (*V*_CHIRP_*D*) voltage responses, we calculated the input (ZAP_N_) and transfer (ZAP_C_) impedance amplitude profiles. Injection of *I*_CHIRP_*S* revealed frequency-dependent attenuation of local input impedance (Z_N_) in TC (1 Hz, 211.3 ± 20.1 MΩ; 20 Hz, 51.2 ± 3.2 MΩ; *n* = 9, *p* < 0.0001, paired *t* test; Fig. 4*A*,*C*) and TRN (1 Hz, 129.3 ± 10.6 MΩ; 20 Hz, 71.6 ± 6.2 MΩ; *n* = 14, *p* < 0.0001, paired *t* test; Fig. 5*A*,*C*) neurons. In our recordings, we did not observe strong membrane potential resonance, and somatic ZAP_N_ in both LT-spiking cells displayed low-pass filter characteristics and could be fit by an RC function. These fits revealed cutoff frequencies (*f*_C_) of 4.9 ± 0.5 and 12.9 ± 0.5 Hz and time constants of 35.0 ± 3.9 and 12.6 ± 0.4 ms in TC (*n* = 9; Fig. 4*C*) and TRN (*n* = 14; Fig. 5*C*) neurons, respectively. In contrast to the soma, and in line with steady-state *R*_N_, the magnitude of dendritic ZAP_N_ in both TC (1 Hz, 524.40 ± 65.76 MΩ; 20 Hz, 341.6 ± 66.41 MΩ; *n* = 9, *p* < 0.0001, paired *t* test; Fig. 4*B*,*D*,*G*) and TRN (1 Hz, 547.8 ± 70.7 MΩ; 20 Hz, 405.0 ± 48.5 MΩ; *n* = 16, *p* < 0.0001, paired *t* test; Fig. 5*B*,*D*,*G*) neurons was dependent on the distance of the recording location from the soma and was also frequency dependent. Dendritic ZAP_N_ could not, however, be fit by the same RC function as somatic ZAP_N_, indicating substantially different dendritic filtering properties. Thus, across a range of frequencies, the distance-dependent somatic and dendritic input impedance profiles matched those observed with DC inputs and were remarkably similar between TC and TRN neurons.

To test whether somatodendritic conduction of non-DC signals in LT-spiking neurons was linear, we calculated ZAP_C_ by dividing the magnitude of voltage response at one recording electrode by that of *I*_CHIRP_ at the other. In both types of LT-spiking cells, ZAP_C_ decreased with increasing signal frequency and, similar to the DC case, was equal in both *S*→*D* and *D*→*S* directions at all frequencies tested [TC (Fig. 4*E–G*), TRN (Fig. 5*E–G*)]. This can be observed by comparing *V*_CHIRP_*D* evoked by *I*_CHIRP_*S* [TC (Fig. 4*A*), TRN (Fig. 5*A*, red trace)] with *V*_CHIRP_*S* evoked by *I*_CHIRP_*D* [TC (Fig. 4*B*), TRN (Fig. 5*B*, blue trace)]. These data demonstrate that somatodendritic conduction of low-frequency (<20 Hz) signals in LT-spiking neurons can be described by a linear model. Furthermore, these findings demonstrate that across a range of input frequencies, the relationship between ZAP_N_, ZAP_C_, and distance from the soma remains constant, and both TC and TRN neurons have distance-dependent dendritic gradients of Z_N_ but nearly spatial uniformity of Z_C_ [TC (Fig. 4*G*), TRN (Fig. 5*G*)]. As a consequence, these properties result in strongly asymmetric voltage transfer in the *S*→*D* and *D*→*S* direction in both TC (Fig. 4*I*) and TRN (Fig. 5*I*). In particular, in the *S*→*D* direction, voltage attenuation is very low and insensitive to input frequency, meaning that current transmission between *D*→*S* remains highly efficient. Accordingly, voltage changes at the soma of LT-spiking neurons are transferred throughout the entire dendritic tree with little reduction in amplitude. This has important consequences for the activation of dendritic T-type Ca^2+^ channels during LTS (discussed below).

We also investigated phase properties by calculating the input (ZPP_N_) and transfer (ZPP_C_) impedance phase profiles. First, as can be seen from ZPP_N_, at all frequencies in both TC (Fig. 4*C*,*D*,*H*) and TRN (Fig. 5*C*,*D*,*H*) neurons, *V*_CHIRP_*S* and *V*_CHIRP_*D* always lagged injected current. Together with the lack of a resonance peak in ZAP_N_, these data suggest that LT-spiking neurons have the properties of a simple low-pass RC electrical circuit. However, both TC and TRN neurons express hyperpolarization-activated cyclic nucleotide-gated channels (HCN; *I*_H_) that are strongly linked to the emergence of resonance and bandpass-filtering properties (Hutcheon and Yarom, 2000). Indeed, some neurons that express *I*_H_ are best modeled not as RC circuits but as RCL circuits (Narayanan and Johnston, 2008; Vaidya and Johnston, 2013). Resonance and bandpass filtering emerges through the interaction between passive and active properties of neurons with resistance (*R*) and capacitance (*C*) acting to passively low-pass (RC) filter signals (*f*_c_ = 1/2πRC), whereas the inductive conductance (*L*) acts as a high-pass (RL) filter (*f*_c_ = 1/2πτ_L_, where τ_L_ equals the channel activation time constant; Hutcheon and Yarom, 2000). In both TC and TRN neurons, as well as other LT-spiking cells including subthalamic neurons and deep cerebellar nucleus neurons, the predominantly expressed channel isoform is HCN-2, which has slow activation kinetics at typical resting membrane potentials. As a consequence, *f*_c_ for the high-pass RL filter in these neurons at rest (−70 mV) is very low (∼0.15 Hz), and thus we see a general broadband suppression of low frequencies up to the low-pass cutoff frequency rather than a sharp resonant-type bandpass profile in the ZAP.

For *I*_CHIRP_*S*, ZPP_N_ was frequency dependent in both TC (Fig. 4*C*) and TRN (Fig. 5*C*) neurons, having minimal phase lag at frequencies <2 Hz (less than −1/12π rad) but phase differences of more than −1/3π radians with 20 Hz input. This corresponds to a time delay of 8–10 ms between the somatic current and voltage (20 Hz). In comparison, although ZPP_N_ for *I*_CHIRP_*D* were also frequency dependent, the phase lag of voltage responses to current input at 20 Hz was significantly less [less than −1/6π rads, 2.5–3 ms; TC (Fig. 4*D*), TRN (Fig. 5*D*)]. Interestingly, dendritic ZPP_N_ showed little change with increasing distance from the soma in both TC (Fig. 4*G*) and TRN (Fig. 5*G*) neurons, indicating that dendritic voltage phase is dictated by local, mostly invariant, membrane properties. Similarly to ZAP_C_, ZPP_C_ calculated as the difference in phase between the local voltage response and current injected at the reciprocal recording site, was frequency dependent and equal in both the *S*→*D* and *D*→*S* direction for TC (Fig. 4*E–H*) and TRN (Fig. 5*E–H*) cells. ZPP_C_ was mostly insensitive to the distance between the voltage recording and current injecting site [TC (Fig. 4*G*), TRN (Fig. 5*G*)]. Thus, in the *S*→*D* direction, *V*_CHIRP_*D* is almost equally phase lagged to *I*_CHIRP_*S* at dendritic recording locations, and in the *D*→*S* direction, *V*_CHIRP_*S* are equally in phase with *I*_CHIRP_*D* regardless of the dendritic input location. This “phase reciprocity” is evident in Figure 4*H* (TC) and Figure 5*H* (TRN) when comparing *V*_CHIRP_*D* (top, red trace) evoked by *I*_CHIRP_*S* with *V*_CHIRP_*S* (bottom, blue trace) evoked by *I*_CHIRP_*D*. As for voltage transfer, these findings demonstrate that phase between somatic and dendritic voltages is highly directionally asymmetric. Thus, in the *S*→*D* direction, phase lag between *V*_CHIRP_*S* and *V*_CHIRP_*D* is minimal at all frequencies when evoked by *I*_CHIRP_*S* in LT-spiking neurons. In comparison, in response to *I*_CHIRP_*D*, we see a significant frequency-dependent phase lag between *V*_CHIRP_*S* and *V*_CHIRP_*D* [TC (Fig. 4*J*), TRN (Fig. 5*J*)]. In summary, these data reveal almost identical somatodendritic transmission properties in two different classes of LT-spiking neurons. Transmission of signals from *S*→*D* and *D*→*S* is highly asymmetric. Thus, current input to the soma, across a range of frequencies, produces voltage changes that are efficiently transmitted throughout the whole dendritic tree with minimal amplitude attenuation and phase shift. Conversely, current input to dendrites produces voltage responses that undergo strong attenuation and have significant phase shifts between the dendrite and soma. As we describe below, these highly conserved dendritic properties are critical in enabling LTS in both TC and TRN neurons.

### Low-threshold spikes cannot be locally generated in dendrites

**Figure 6.**
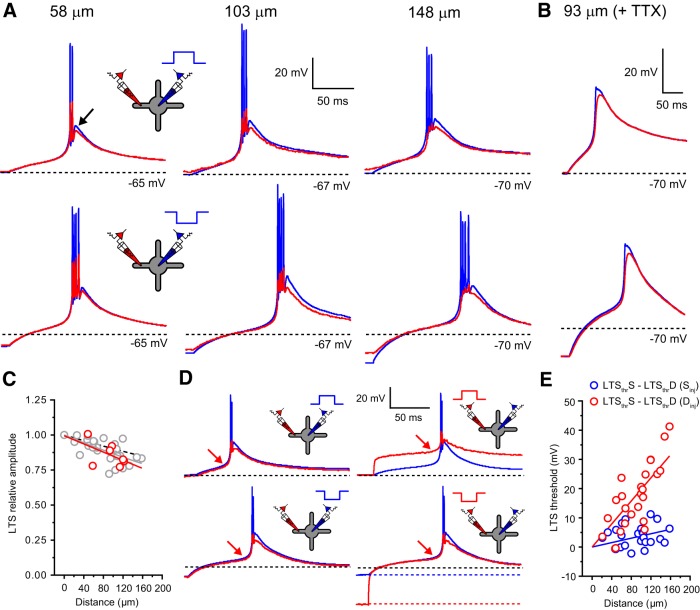
Low-threshold spikes cannot be generated locally in TC neuron dendrites. ***A***, LTS_S_ (blue) and LTS_D_ (red) evoked by depolarizing (top) and hyperpolarizing (bottom) somatic current injection steps. Data are depicted from three separate TC neurons at the indicated dendritic recording locations. The dashed black line indicates resting membrane potential. The black arrow indicates the peak of the Ca^2+^ spike used to measure LTS amplitude. ***B***, As in ***A*** for a single TC neuron in the presence of TTX (0.5 μm). ***C***, Plot of LTS_D_ amplitude normalized to LTS_S_ amplitude versus distance from soma. Gray circles, LTS_D_ (−TTX); solid gray line, exponential fit to normalized LTS_D_ amplitudes (−TTX); red circles, LTS_D_ (+TTX); solid red line, exponential fit to normalized LTS_D_ amplitudes (+TTX); dashed black line, steady-state *S*→*D* voltage attenuation. ***D***, LTS_S_ (blue) and LTS_D_ (108 μm, red) evoked by depolarizing (top) and hyperpolarizing (bottom) current injection steps into either the soma (left) or dendrite (red). The dashed black line indicates resting membrane potential. The red arrow indicates LTS threshold (δ*V*/δ*t* > 0.5 mV · ms^−1^). ***E***, Plot of LTS voltage threshold versus distance from the soma. Blue circles. Difference in LTS_S_ and LTS_D_ threshold when evoked by somatic current injection; red circles, difference in LTS_S_ and LTS_D_ threshold when evoked by dendritic current injection.

**Figure 7.**
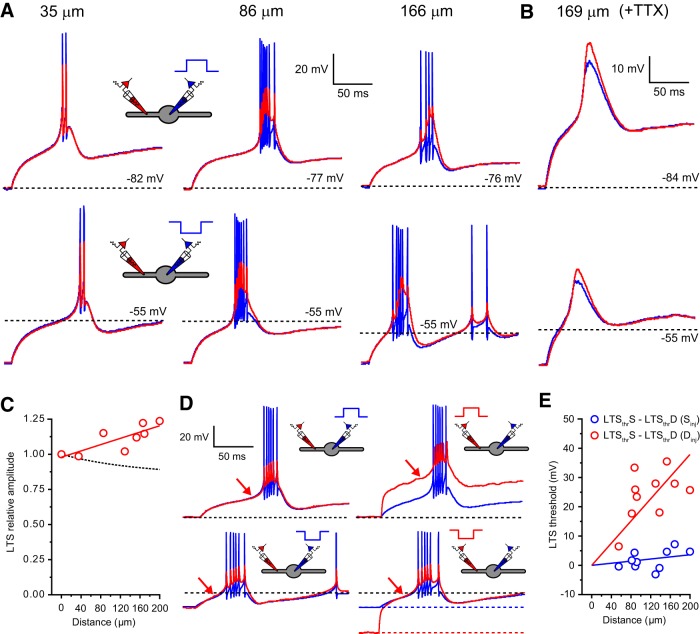
Low-threshold spikes cannot be generated locally in TRN neuron dendrites. ***A***, LTS_S_ (blue) and LTS_D_ (red) evoked by depolarizing (top) and hyperpolarizing (bottom) somatic current injection steps. Data are depicted from three separate TRN neurons at the indicated dendritic recording locations. The dashed black line indicates resting membrane potential. ***B***, As in ***A*** for a single TRN neuron in the presence of TTX (0.5 μm). ***C***, Plot of LTS_D_ amplitude normalized to LTS_S_ amplitude versus distance from soma. Red circles, LTS_D_ (+TTX); solid red line, exponential fit to normalized LTS_D_ amplitudes (+TTX); dashed black line, steady-state *S*→*D* voltage attenuation. ***D***, LTS_S_ (blue) and LTS_D_ (108 μm, red) evoked by depolarizing (top) and hyperpolarizing (bottom) current injection steps into either the soma (left) or dendrite (red). Dashed black line, Resting membrane potential; red arrow, LTS threshold (δ*V*/δ*t* > 0.5 mV · ms^−1^). ***E***, Plot of LTS voltage threshold versus distance from the soma. Blue circles, Difference in LTS_S_ and LTS_D_ threshold when evoked by somatic current injection; red circles, difference in LTS_S_ and LTS_D_ threshold when evoked by dendritic current injection.

Our data reveal that despite differences in dendritic morphology (Fig. 2*A*, and Fig. 3*A*), major T-type Ca^2+^ channel subunit expression (mostly Ca_V_3.1 in TC cells and a combination of Ca_V_3.2 and 3.3 in TRN cells; Perez-Reyes, 2003), and neurotransmitter released (glutamate/GABA), the two classes of LT-spiking neurons investigated here have remarkably similar dendritic electrical properties. We hypothesized that, in combination with dendritic expression of T-type Ca^2+^ channels (Crandall et al., 2010; Errington et al., 2010), these highly conserved properties would be critical to the most prominent common feature of these cells, the LTS. Thus, we tested which of three proposed models (somatic, dendritic, or global initiation) best explains generation and spread of LTS in LT-spiking neurons. First, we simply examined LTS amplitudes in the soma (LTS_S_) and dendrites (LTS_D_) of TC and TRN neurons. In TC neurons, the amplitude of LTS could be measured in the presence of Na^+^-mediated action potentials as the difference between the peak Ca^2+^ spike (Fig. 6*A*, black arrow) and the membrane potential before the current injection step. This was not possible in TRN neurons, owing to the structure of their LTS (Fig. 7*A*), and quantitative data were therefore obtained from recordings performed in the presence of TTX (0.5 μm). In TC cells, when evoked by either depolarizing or hyperpolarizing somatic current injection, LTS_D_ showed minimal reduction in amplitude relative to their somatic counterparts (0.72–1.00, 0.89 ± 0.01, *n* = 28; Fig. 6*A*,*C*) but did depend on distance from the soma (λ_eff_ = 697 μm, *n* = 28). In these neurons, differences in the amplitude of LTS_S_ and LTS_D_ in the presence of bath-applied TTX were not significantly different from those measured under control conditions (0.5 μm; 0.77–1.01, 0.86 ± 0.03, *n* = 7, *p* = 0.38, unpaired *t* test; Fig. 6*A*,*C*). In fact, the difference in amplitude between LTS_S_ and LTS_D_ in TC neurons was similar to (VS_S_→VD_S_) (Fig. 6*C*), suggesting, perhaps, that LTS might originate in the soma and propagate into dendrites. However, in TRN neurons, we found that LTS_D_ increased in amplitude relative to LTS_S_ with increasing distance from the soma (0.99–1.24, 1.11 ± 0.03, *n* = 8), in opposition to (VS_S_→VD_S_) in these cells (Fig. 7*A–C*). These data appear inconsistent with a somatic origin and support LTS generation in the dendrites of LT-spiking cells (see below). Consequently, we tested whether LTS are initiated in dendrites of LT-spiking cells using dendritic current injection steps of increasing size. Evoking LTS using somatic current injection revealed only minimal difference in the voltage threshold (δ*V*/δ*t* > 0.5 mV · ms^−1^) at which LTS occurred in the soma and dendrites of both TC (−2.2 to 11.2 mV, 3.7 ± 0.7 mV, *n* = 25; Fig. 6*D*,*E*) and TRN (−3.1 to 7.2 mV, −1.9 ± 1.0 mV, *n* = 10; Fig. 7*D*,*E*) neurons. It is important to note that in this case, the threshold refers to the apparent membrane potential in the dendrite at which LTS can be evoked and does not refer to the activation threshold for T-type Ca^2+^ channels that we expect to be the same at both the soma and in the dendrites. In fact, in our computational model, no differences in activation or inactivation properties of T-type Ca^2+^ channels between soma and dendrites were required to reproduce the voltage responses we recorded experimentally. In clear comparison with somatic input, when evoked by dendritic current injection, a pronounced distance-dependent difference in the threshold for LTS_S_ and LTS_D_ in both TC (−0.5 to 41.3 mV, 17.1 ± 2.2 mV, *n* = 25; Fig. 6*D*,*E*) and TRN (6.5–35.5 mV, 24.20 ± 2.7 mV, *n* = 10; Fig. 7*D*,*E*) neurons was observed. The distance-dependent difference between dendritic and somatic threshold reflects the inefficient VD_D_→VS_D_ in these cells. As a consequence, dendritic voltage change, whose amplitude would be comfortably suprathreshold for LTS generation if occurring at the soma, was incapable of triggering “local” dendritic LTS. This does not imply that local dendritic T-type Ca^2+^ channels do not contribute to local sub-LTS threshold voltage changes but demonstrates that they are not sufficient to trigger an all-or-none local LTS without activation of downstream channels in the soma and contralateral dendrites. As demonstrated in Figures 6*D* and 7*D*, the minimal dendritic depolarization or hyperpolarization required to evoke an LTS was substantially greater than that required at the soma. On the other hand, the threshold voltage for LTS_S_ was equal for both somatic and dendritic current injections in TC (soma, −59.95 ± 0.46 mV; dendrite, −58.61 ± 0.87 mV; *n* = 25, *p* = 0.22, unpaired *t* test) and TRN (soma, −57.14 ± 1.13 mV; dendrite, −59.29 ± 0.82 mV; *n* = 10, *p* = 0.14, unpaired *t* test) neurons. Furthermore, the threshold for LTS_D_, evoked by a large hyperpolarizing dendritic current injection, was the same as LTS_S_ but substantially lower than the threshold for LTS_D_ evoked by depolarizing steps (Figs. 6*D*, 7*D*).

In fact, the ZAP and ZPP data suggest that, even with an active contribution from dendritic T-type Ca^2+^ channels, a much higher degree of amplitude attenuation and phase lag would be expected for a *D*→*S* propagating signal than we observed between LTS_S_ and LTS_D_ (Fig. 6*B*, 7*B*). Consequently, we conclude that LTS cannot be initiated in dendrites, and to trigger an LTS, dendritic input must be sufficient to depolarize the soma to LTS threshold.

### Low-threshold spikes are generated by a globally distributed mechanism

**Figure 8.**
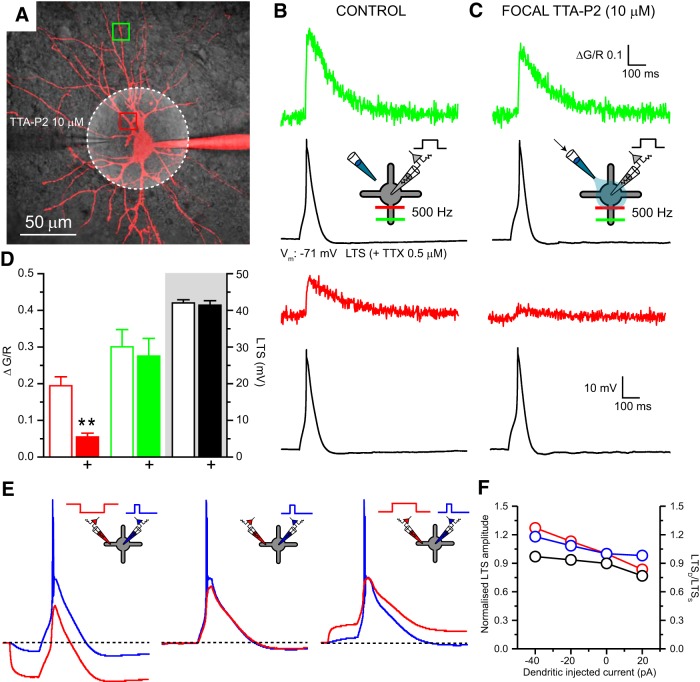
Dendritic T-type Ca^2+^ channels have an active role in low-threshold spike generation. ***A***, Overlay of two-photon fluorescence and infrared scanning gradient contrast images of a dLGN TC neuron showing location of somatic recording (red) and TTA-P2 application pipettes. Dashed circle, Approximate spread of pressure-applied TTA-P2 (10 μm); red box, region of interest for proximal line scan; green box, region of interest for distal line scan. ***B***, Δ[Ca^2+^] in proximal (red) and distal (green) dendrites produced by a somatically evoked LTS (black) under control conditions (+TTX, 0.5 μm). ***C***, Δ[Ca^2+^] at the same dendritic locations produced by a somatically evoked LTS during concurrent focal pressure application of TTA-P2. Insets depict the experimental configuration. ***D***, Plot showing the significant reduction in mean proximal (red, *p* < 0.01, *n* = 4) but not distal (green, *p* > 0.05, *n* = 4) Δ[Ca^2+^] and lack of effect on somatically recorded LTS (black) produced by pressure-applied focal TTA-P2. ***E***, LTS_S_ (blue) and LTS_D_ (red) evoked by somatic current injection during different levels of concurrent dendritic depolarizing (+20 pA) or hyperpolarizing (−40 pA) current injection. ***F***, Plot summarizing the effects of dendritic polarization on the amplitude of LTS_S_ (blue circles) and LTS_D_ (red circles) normalized to their amplitude when evoked from rest (0 pA) and the ratio of LTS_D_-to-LTS_S_ (black circles).

Having discounted local dendritic LTS initiation, we examined which of the remaining hypotheses, namely local somatic or global initiation, is responsible for generating LTS. Since it is highly improbable that, even in the presence of active conductances, a spike originating in the soma would increase in amplitude as it propagated into the higher-impedance dendritic tree, the amplitude of LTS_D_ versus LTS_S_ in TRN neurons, coupled with a clear inability to generate LTS in dendrites, strongly suggests a global mechanism. However, in TC neurons, the relative amplitudes of LTS_D_ and LTS_S_ are consistent with both a global or local somatic spike generation mechanism. Therefore, we focused on LTS generation specifically in TC neurons. First, we reasoned that if they were triggered at the soma, LTS could be blocked by selective inhibition of only somatic T-type Ca^2+^ channels. To test this, we combined electrical recordings, two-photon Ca^2+^ imaging, and focal application of the selective T-type Ca^2+^ channel antagonist TTA-P2 (Dreyfus et al., 2010). In the presence of bath-applied TTX (0.5 μm) calcium transients (Δ[Ca^2+^]), measured in proximal (<20 μm; Δ*G*/*R*, 0.19 ± 0.02; *n* = 4) and distal (>120 μm; Δ*G*/*R*, 0.30 ± 0.05; *n* = 4) dendrites of TC neurons (Fig. 8*A*,*B*,*D*) during an LTS evoked by somatic current injection (42.0 ± 0.9 mV, *n* = 4), were similar to those described previously (Errington et al., 2010). Focal application of TTA-P2 (10 μm; Fig. 8*A*), at a concentration sufficient to easily block all T-type Ca^2+^ channels (Dreyfus et al., 2010), resulted in ∼75% reduction in the size of proximal (0.05 ± 0.1, *n* = 4, *p* = 0.0027, paired *t* test) but not distal (0.28 ± 0.05, *n* = 4, *p* = 0.46, paired *t* test) Δ[Ca^2+^] (Fig. 8*C*,*D*). Nonetheless, in the presence of TTA-P2, the somatically recorded LTS remained unaffected (41.4 ± 1.2 mV, *n* = 4, *p* = 0.47, paired *t* test; Fig. 8*B–D*). Thus, somatic T-type Ca^2+^ channels are not a necessary requirement for LTS generation. These data offer support to the hypothesis that LTS are generated not by a focal but global mechanism requiring T-channels throughout the dendritic tree.

The involvement of dendritic T-type Ca^2+^ channels in LTS generation was supported by dendritic recording experiments. We surmised that if LTS_D_ passively reflects LTS_S_, without an active dendritic T-type Ca^2+^ channel contribution, the ratio of LTS_D_ and LTS_S_ amplitude (LTS_D_/LTS_S_) would be insensitive to dendritic polarization. However, evoking LTS by brief somatic current injection during concurrent injection of long current steps into dendrites (93–122 μm from the soma) revealed that dendritic hyperpolarization (−40pA, 0.97 ± 0.02; *n* = 5, *p* > 0.05, repeated-measures ANOVA) reduces and depolarization (+20 pA, 0.77 ± 0.03; *n* = 5, *p* < 0.05, repeated-measures ANOVA) increases the difference in amplitude between LTS_D_ and LTS_S_ compared with rest (0 pA, 0.90 ± 0.03, *n* = 5; Fig. 8*E*,*F*). Moreover, these experiments reveal that even dramatic membrane polarization cannot induce local failure of LTS in dendrites, providing additional support for the global generation hypothesis.

**Figure 9.**
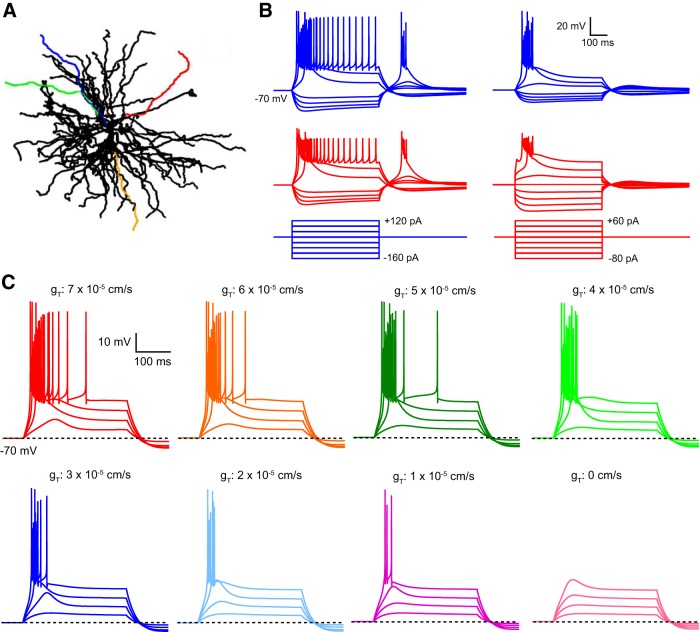
Somatic and dendritic properties of our TC neuron computational model. ***A***, Schematic illustration of the morphology of the model cell. Color-coded dendrites were selected based on their local *R*_N_. For most experiments, simulated dendritic data were taken at the midpoint of the red dendrite. ***B***, Simulated somatic (blue) and dendritic (red) responses to current injection into either the soma or dendrite of the model TC neuron. These simulated responses are recorded from the model cell under standard conditions (*g*_T_, 7 × 10^−5^ cm/s). ***C***, Simulated LTS evoked by somatic current steps of 25–100 pA with *g*_T_ in the dendrites set to different levels.

**Figure 10.**
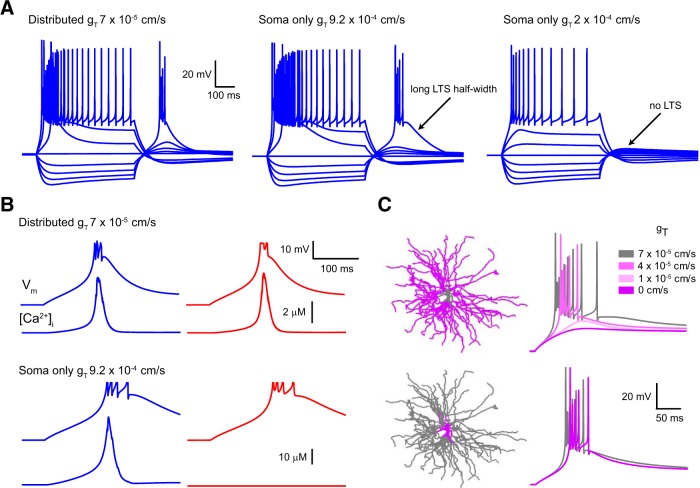
Uniform distribution of dendritic T-type Ca^2+^ channels allows global LTS generation. ***A***, Simulated voltage responses to somatic current injections (40 pA steps) in the TC model cell with varying distribution and density of T-type Ca^2+^ channel conductance. The uniform distribution of conductance most accurately reproduces the voltage response observed in experiments. With soma-only placement of channels, significantly higher local conductance was required (9.2 × 10^−4^ cm/s) to produce LTS. With this channel distribution, note the unusually long LTS duration compared with those measured experimentally. Soma-only channels with a density 10-fold greater (2 × 10^−4^ cm/s) than the minimum distributed density required to produce an LTS were unable to produce an LTS. ***B***, Uniform distribution of T-type Ca^2+^ channels results in similar-sized Δ[Ca^2+^] in the soma (blue) and dendrites (red), consistent with previous Ca^2+^ imaging data. Bottom traces show the Δ[Ca^2+^] produced with only somatic T-type Ca^2+^ channels. No increase in dendritic Ca^2+^ is observed with this channel distribution. ***C***, Model LTS with varying T-type Ca^2+^ channel density in somatic and dendritic regions. Schematic representations of the model cell show the regions where T-type Ca^2+^ channel conductance was altered (purple). Systematically reducing *g*_T_ in the dendrites resulted in LTS failure. Reducing *g*_T_ to 0 cm/s in the soma and proximal dendrites had a minimal effect on the LTS.

Nonetheless, although dendritic T-type Ca^2+^ channels appear to be actively involved in LTS generation and are sufficient in the absence of somatic channels to generate the LTS, a key question remains. Is the somatic T-channel density alone sufficient to locally initiate an LTS? Since it is experimentally implausible to selectively block dendritic T-channels while allowing somatic channels to remain available, to answer this question we developed a new multicompartment computational model of a TC neuron based on our novel dendritic recordings and morphology taken from Briska et al. (2003). Results from four dendrites (Fig. 9*A*), selected to encompass a range of branching patterns and local input impedances, demonstrate that our model accurately reproduces the “passive” cellular properties we observed experimentally, including asymmetric voltage transfer, somatodendritic *R*_N_ gradient, and uniform *R*_c_ distribution (Fig. 1*E–G*). Subsequently, to model the LTS, we tested several T-type Ca^2+^ channel distributions including somatic-only, graded *S*→*D*, graded *D*→*S*, and uniform distribution. Uniform distribution of T-type Ca^2+^ channel conductance (*g*_T_) produced model voltage responses in soma and dendrites most similar to those seen in dual patch-clamp recordings (Fig. 9*B*) while also reproducing intracellular Δ[Ca^2+^] equivalent to those described previously (Fig. 10*B*; Crandall et al., 2010; Errington et al., 2010, 2012; Sieber et al., 2013). Moreover, the range of uniformly distributed *g*_T_ densities capable of reproducing the voltage signals we recorded *in vitro* was relatively narrow, and the optimal value (*g*_T_, 7 × 10^−5^ cm/s) was similar to that reported in a previous study (Destexhe et al., 1998; Fig. 9*C*).

Thus, using these parameters, our TC neuron model could reproduce both the electrical signals and Δ[Ca^2+^] observed in the soma and dendrites during LTS (Fig. 10*A*,*B*). First, consistent with our previous experimental data (Fig. 8), we found that when *g*_T_ was uniformly distributed, its complete removal from the soma and proximal (≤20 μm from the soma) dendrites alone had almost no effect on LTS generation (Fig. 10*C*). In marked contrast, when the level of *g*_T_ was systematically and selectively reduced only in dendrites (>20 μm from the soma), we found a progressive reduction in the ability to produce LTS (Fig. 10*C*). Interestingly, we found that a reduction in dendritic *g*_T_ of ∼70% (∼1–2 × 10^−5^ cm/s) was required to preclude LTS generation in our model (Fig. 9*C*), a finding that mimics previous *in vitro* data demonstrating a level of “channel redundancy” for LTS generation in LT-spiking neurons with uniform T-channel distribution (Dreyfus et al., 2010). Thus, even though the density of somatic *g*_T_ (7 × 10^−5^ cm/s) in our standard model conditions was somewhat higher than that estimated in the soma of dissociated TC neurons (1.7 × 10^−5^ cm/s; Destexhe et al., 1998), in the absence of dendritic T-channels, our model indicates that somatic channels alone are incapable of generating a local LTS. Finally, to test whether the inability of somatic channels alone to produce an LTS under our standard model conditions was attributable to an underestimate of somatic *g*_T_, we concentrated the total conductance from our uniform model into the soma alone (*g*_T_, 9.2 × 10^−4^ cm/s). Whereas this was able to produce LTS in both soma and dendrites of our model TC neuron (Fig. 10*A*,*B*), these potentials differed significantly from those we recorded experimentally and produced markedly different Δ[Ca^2+^] distributions from those observed experimentally in these dendrites (Fig. 8*B*; Crandall et al., 2010; Errington et al., 2010, 2012; Sieber et al., 2013). Furthermore, similarly to the distributed case, although a 70% reduction in the density of soma-only *g*_T_ was required to prevent LTS generation, the resulting *g*_T_ density (2 × 10^−4^ cm/s) at which LTS failed (Fig. 10*A*) remained an order of magnitude greater than both that estimated at the soma experimentally (Destexhe et al., 1998) and the minimum density of uniformly distributed *g*_T_ (*g*_T_, 2 × 10^−5^ cm/s) capable of producing LTS in our model (Fig. 9*C*).

Consequently, we conclude that LTS cannot be locally generated at the soma of LT-spiking neurons. Based on our data, we propose that LTS are produced by a unique mechanism that exploits the dendritic electrical properties of LT-spiking neurons to allow the synchronous recruitment of widely spatially distributed T-type Ca^2+^ channels. These relatively low-density channels act in concert to produce a global LTS. Finally, although TC neurons predominantly express Ca_V_3.1 (α1G) T-type Ca^2+^ channel subunits and TRN cells express both Ca_V_3.2 (α1G) and Ca_V_3.3 (α1I; Perez-Reyes, 2003), both cells generate LTS using the same globally distributed mechanism. Thus, whereas the precise molecular identity of the T-type Ca^2+^ channel subunits expressed in particular neurons might shape their LTS properties, specific expression patterns of particular isoforms are not required for global spike generation as long as T-type Ca^2+^ channels are roughly uniformly expressed throughout the somatodendritic tree.

## Discussion

The major findings of this study are that (1) dendritic properties are highly conserved between two prominent types of LT-spiking cells despite differences in their morphology and function; (2) these properties play a critical role in LTS generation by enabling synchronous recruitment of spatially distributed dendritic T-type Ca^2+^ channels; and (3) this underlies a unique whole-cell Ca^2+^ spiking mechanism that sets the LTS apart as a distinctive, all-or-none, global somatodendritic electrical and biochemical signal. We propose that this mechanism applies to all LT-spiking cells and is likely to have important implications for synaptic signaling, dendritic integration, and plasticity.

### Conserved dendritic properties in LT-spiking neurons

The dendritic properties of LT-spiking neurons have not previously been studied directly, despite evidence of a role for dendritic T-type Ca^2+^ channels in LTS generation (Destexhe et al., 1998; Williams and Stuart, 2000; Errington et al., 2010). Here, we find that key features of dendritic electrical conduction are highly conserved between TC and TRN cells. In particular, our data reveal that signal transfer between soma and dendrites in these neurons is strongly directionally asymmetric. Thus, whereas (VS_S_→VD_S_) is particularly efficient, VD_D_→VS_D_ is much more inefficient. We find that, rather than through loss of current from leaky dendrites, this marked disparity occurs as a result of drastic increases in local dendritic Z_N_ with increased distance from the soma. Consequently, as the distance between two points along the somatodendritic axis increases, there is little variation in Z_C_. This means that the efficiency of current transmission from dendrites to the soma of LT-spiking neurons is virtually independent of the dendritic input location. In a previous study, Jaffe and Carnevale (1999) demonstrated that in neurons with this somatodendritic profile of Z_N_ and Z_C_, synaptic currents can produce nearly equal somatic EPSPs independently of their position within the dendritic tree; a process they termed “passive normalization.” Our findings indicate that synaptic passive normalization may be an important feature of signaling in LT-spiking neurons. In TC and TRN neurons, this mechanism could allow corticothalamic synapses, which are distributed widely across the dendritic tree, to “democratically” exert an analog-like control over resting membrane potential and permit a smooth rapid transition between the burst and tonic firing modes that are characteristic of these cells.

### The global low-threshold spike

Our new findings illustrate fundamental differences between the LTS and other neuronal spikes. Thus, whereas action potentials are initiated by high-density Na^+^ channels in the axon initial segment (Kole et al., 2008; Foust et al., 2010) and dendritic Ca^2+^ (Schiller et al., 1997; Larkum et al., 2009) and NMDA (Schiller et al., 2000; Larkum et al., 2009) spikes are locally triggered in individual dendritic branches by voltage-gated Ca^2+^ channels or “hot spots” of clustered NMDA receptor conductance in LT-spiking cells, because T-type Ca^2+^ channel density is insufficient to produce local spikes, LTS generation relies on activation of distributed dendritic T-type Ca^2+^ conductances. Consequently, LTS do not propagate in a traditional manner from a focal initiation zone to the rest of the cell but instead occur throughout the soma and dendrites simultaneously. The global nature of the LTS sets it apart from other electrical signaling mechanisms that typically exert graded effects throughout the somatodendritic tree and are often locally isolated to specific subcellular compartments (e.g., individual dendritic branches; Larkum et al., 2009).

Global LTS generation can be understood by considering the electrotonic structure of LT-spiking neurons. In particular, since we find that *S*→*D* voltage transfer is highly efficient with minimal phase shift, LT-spiking neurons are extremely electrotonically compact in the *S*→*D* direction. Therefore, although dendritic T-type Ca^2+^ channels may be separated in physical space by several hundred micrometers (e.g., at the tips of opposing TRN neuron dendrites), in electrotonic space they are much closer together because the complex, branching dendritic trees of LT-spiking neurons are effectively collapsed to allow the cell to behave more like an iso-potential sphere. Thus, as the soma is depolarized (e.g., by summating synaptic potentials), the membrane potential in even the most distal regions of the dendritic tree follows with minimal difference in phase or amplitude. This means that the relatively low-density dendritic T-type Ca^2+^ channels can be activated together and act in concert to produce an LTS.

From this model, it is clear that the voltage recorded at any point on an LT-spiking cell during an LTS relies more on a global depolarizing effect produced by summed distal current sources than depolarization resulting from local current flow. Supporting this idea, to trigger an LTS, dendritic current injection sufficient to depolarize the soma and, as a consequence of efficient (VS_S_→VD_S_), the “downstream” dendritic tree to spike threshold was required. As such, LTS only occur under conditions where the entire cell is depolarized to LT-spiking threshold. Even strong dendritic depolarization cannot produce LTS if the resulting somatic potential does not reach spike threshold because local T-type Ca^2+^ conductance is too low to permit spike initiation. These data show that, as well as producing Ca^2+^ signals (Crandall et al., 2010; Errington et al., 2010, 2012; Sieber et al., 2013) throughout LT-spiking cells, LTS are always associated with a global electrical signal whose amplitude and phase are mostly independent of where they are recorded within the cell.

Another explanation for the results we present here is that LTS are generated by a high-density hot spot of T-type Ca^2+^ channels in the axon, a structure that, for technical reasons, we are unable to image or directly record from in these neurons. Several strong lines of evidence argue against this mechanism. First, the dendritic Δ[Ca^2+^] we observed in our computational model, with uniform somatodendritic distribution of T-type Ca^2+^, were very similar to those previously described experimentally (Errington et al., 2010). This channel distribution accurately reproduced the somatic and dendritic membrane potential transients we recorded without requiring a focal hot spot of T-type Ca^2+^ channels in the axon or elsewhere. Second, in both TRN neurons and cortical LT-spiking interneurons, there is no evidence for an axonal hot spot of T-type calcium channels, and instead these channels are found to be distributed across the dendritic membrane (Liu et al., 2011). Third, in experiments where we focally applied the T-type Ca^2+^ antagonist TTA-P2 to TC neurons, we saw no difference in the somatically recorded LTS. In these experiments, we would also expect to block any T-type Ca^2+^ channels in the initial segment of the axon. Consequently, these data suggest that it is unlikely that the LTS is generated by an axonal mechanism and provide support to the whole-cell mechanism we propose.

LT-spiking neurons share a number of important similarities including similar morphologies with long thin dendrites that are relatively aspiny. Specifically, LT-spiking cells lack large apical dendrites, and as such, their dendritic morphology is compatible with them having electrical conduction properties similar to those we have reported for TC and TRN neurons (Jaffe and Carnevale, 1999). LT-spiking neurons also have similar somatically recorded electrical characteristics, in particular the ability to generate rebound LTS. Furthermore, in common with TC and TRN neurons (Crandall et al., 2010; Errington et al., 2010), both low-threshold spiking cortical interneurons (Goldberg et al., 2004) and subthalamic neurons (Atherton et al., 2010) have LTS-evoked T-type Ca^2+^ channel-dependent dendritic Δ[Ca^2+^]. Therefore, although recording from dendrites of all LT-spiking neuron dendrites is beyond the scope of a single study, we anticipate that LTS in all LT-spiking cells result from the global mechanism we have described here.

### Physiological consequences of global low-threshold spikes

Whereas our new findings explain how LTS are generated, they do not answer why a global distribution of T-type Ca^2+^ channels is favored. In TC neurons, one reason might relate to the fact that action potential bursts associated with low-threshold spikes more strongly activate their neocortical targets than single action potentials (Swadlow and Gusev, 2001). As such, it is thought that this wake-up call represents a critical form of thalamic signaling to cortex. Since LTS produce a global electrical and biochemical signal throughout the entire dendritic tree, all synapses would be able to “sense” the occurrence of an LTS regardless of its location. Thus, in this case, the LTS acts as an intracellular synchronization signal that couples this special form of TC output to the site of synaptic input in the dendrites, perhaps to reset dendritic integration or induce synaptic plasticity. Related to this point, it has been demonstrated that LTS play a critical role in LTP at inhibitory TRN neuron synapses on to TC neurons by producing Ca^2+^ entry through both T- and L-type Ca^2+^ channels (Sieber et al., 2013). In this situation, because of its all-or-none nature, the LTS could act as a global, somatodendritic plasticity signal causing synaptic potentiation at all TRN synapses rather than targeting specific inputs. This may indicate a homeostatic plasticity role for the LTS that helps to stabilize neurons and networks within particular states (e.g., through Ca^2+^-dependent enhancement of HCN channels; Lüthi and McCormick, 1998) to alter oscillatory bursting during sleep.
